# Carbon turnover in the water-soluble protein of the adult human lens

**Published:** 2013-02-25

**Authors:** Daniel N. Stewart, Jozsef Lango, Krishnan P. Nambiar, Miranda J. S. Falso, Paul G. FitzGerald, David M. Rocke, Bruce D. Hammock, Bruce A. Buchholz

**Affiliations:** 1 Department of Chemistry, University of California, One Shields Avenue, Davis, CA; 2 Center for Accelerator Mass Spectrometry, Lawrence Livermore National Laboratory, 7000 East Avenue, Livermore, CA; 3 Department of Cell Biology and Human Anatomy, School of Medicine, University of California, Davis, CA; 4 Division of Biostatistics, School of Medicine, University of California, Davis, CA; 5 Department of Entomology and Comprehensive Cancer Center, University of California, One Shields Avenue, Davis, CA; 6 Currently Division of Math and Natural Sciences, Pennsylvania State University – Altoona, Altoona, PA

## Abstract

**Purpose:**

Human eye lenses contain cells that persist from embryonic development. These unique, highly specialized fiber cells located at the core (nucleus) of the lens undergo pseudo-apoptosis to become devoid of cell nuclei and most organelles. Ostensibly lacking in protein transcriptional capabilities, it is currently believed that these nuclear fiber cells owe their extreme longevity to the perseverance of highly stable and densely packed crystallin proteins. Maintaining the structural and functional integrity of lenticular proteins is necessary to sustain cellular transparency and proper vision, yet the means by which the lens actually copes with a lifetime of oxidative stress, seemingly without any capacity for protein turnover and repair, is not completely understood. Although many years of research have been predicated upon the assumption that there is no protein turnover or renewal in nuclear fiber cells, we investigated whether or not different protein fractions possess protein of different ages by using the ^14^C bomb pulse.

**Methods:**

Adult human lenses were concentrically dissected by gently removing the cell layers in water or shaving to the nucleus with a curved micrometer-controlled blade. The cells were lysed, and the proteins were separated into water-soluble and water-insoluble fractions. The small molecules were removed using 3 kDa spin filters. The ^14^C/C was measured in paired protein fractions by accelerator mass spectrometry, and an average age for the material within the sample was assigned using the ^14^C bomb pulse.

**Results:**

The water-insoluble fractions possessed ^14^C/C ratios consistent with the age of the cells. In all cases, the water-soluble fractions contained carbon that was younger than the paired water-insoluble fraction.

**Conclusions:**

As the first direct evidence of carbon turnover in protein from adult human nuclear fiber cells, this discovery supports the emerging view of the lens nucleus as a dynamic system capable of maintaining homeostasis in part due to intricate protein transport mechanisms and possibly protein repair. This finding implies that the lens plays an active role in the aversion of age-related nuclear (ARN) cataract.

## Introduction

The overall structure of the human lens is one of successive generations of secondary fiber cells stratified chronologically around the embryonic nucleus, with the primary fiber cells being formed by week six after fertilization [1,2]. Because all cells are retained, the lens grows continuously throughout life, and its history is well preserved within the tissue.

The formation of age-related nuclear (ARN) cataracts is associated with the aging of the lens and characterized by the loss of optical transparency [3]. Age is the single largest risk factor for ARN cataract; however, there are clear distinctions between the protein alterations brought on by the aging process and those associated with cataract formation [4-10]. ARN cataracts are typically associated with oxidative stress, which causes conformational changes, aggregation, and the loss of solubility of the abundant, densely packed proteins of the lens fiber cells [1,3,4,11,12]. The lens must support the functionality of all of its constituent cells for a lifetime, yet the manner by which it preserves homeostasis in its aged nuclear fiber cells is not fully understood. It is presumed that the failure to preserve the viability of any fiber cell leads to pathology [11].

An “inner circulatory system” of ion channels, Na/K pumps, gap junctions, and water channels facilitates the active transport of fluid, ions, and small molecules between the superficial and innermost regions of the lens to maintain homeostasis [13-18]. Aquaporin-0 (AQP0), or membrane intrinsic protein, is the primary aquaporin expressed in lens fibers. Aquaporins are integral membrane proteins with the main function of transporting water across cell membranes. Mutations in AQP0 have been shown to play a role in ARN cataract formation [19,20]. Compared to other aquaporins, AQP0 demonstrates low water permeability, and it has been proposed that AQP0 plays other roles that may relate to ARN cataract formation. A variety of age-related post-translational modifications have been identified in AQP0, but how these modifications pertain to the formation of ARN cataracts has not been determined [21]. A second group of membrane proteins, the connexins (Cx), interacts with AQP0 to form the fiber-to-fiber junctions [22]. Cataracts often develop as a secondary effect of type II diabetes, and when a cataract lens from one of these patients was examined, Cx were lacking from the lens and the AQP0 arrays were misformed, leading to a breakdown of the lens microcirculation system and opacification [16,23]. Mutations to Cx are a common cause of hereditary cataracts [16]. AQP0 also acts as a scaffold protein that organizes γ-crystallins [24]. Crystallins are the major protein component of the lens, with α-crystallin acting as a molecular chaperone and scaffold protein, and β- and γ-crystallins as structural proteins [3]. A colloidal model of α- and γ-crystallin proteins demonstrated another proposed mechanism for the formation of ARN cataracts. A weak, short-range attraction between α- and γ-crystallins maintains the transparency of concentrated crystallin mixtures, and changes in the magnitude of this interaction may contribute to the formation of ARN cataracts [25]. In addition, an aging lens may experience adhesion of α-, β-, and γ-crystallins or fragments thereof to cell membranes, which may contribute to a loss in lens flexibility and the inhibition of membrane properties [8,9]. These studies all demonstrate the necessity of a functional circulatory system in the lens in order to prevent cataract formation.

A correlation has been found between extensive protein oxidation, which is the hallmark of age-related nuclear (ARN) cataract, and the barriers to metabolite transport that develop between the cortical and nuclear regions of the lens in middle age [4,26]. In addition to the “inner circulatory system” described above for the transport of fluids, ions, and low molecular mass constituents, research investigating large molecule transport has indicated a protein permeable pathway within the nuclei of chicken and mouse lenses and demonstrated how those nuclei behave as a syncytium [27-29]. More than forty years ago, it was shown that the ^35^S-methionine crossed the cortical boundary in rats and entered the lens nucleus, but autoradiography could not determine whether the ^35^S-methionine was free or incorporated in lens crystallin proteins [30]. Possibly even more striking was the discovery of significant numbers of transcriptionally capable fiber cells located below the cortical-nuclear barrier in an adult population of mice [28]. Although these studies have shown that protein generation and transport is possible in the mammalian lens, most researchers still tacitly assume that protein turnover is nonexistent in human nuclear fiber cells, for there have been no long-term metabolic labeling studies to evaluate this phenomenon. Circumventing the time constraints and feasibility of performing such a lengthy trial involving human subjects, we describe a “retrospective tracer” study in which we aim to determine carbon turnover in the protein of aged cells without the need for the administration of chemical tracers to the subjects before the analysis.

**Figure 1:**
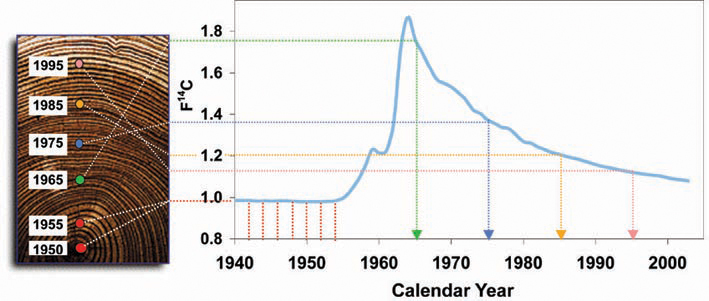
The ^14^C bomb curve is recorded in biomolecules. Aboveground nuclear testing nearly doubled the level of radiocarbon (as ^14^CO_2_) in the atmosphere between 1955 and 1963. The atmospheric ^14^C levels depicted by the dark blue trace are growing season averages for the northern hemisphere expressed in F^14^C units (fraction modern with δ^13^C fractionation correction). These ^14^C levels are recorded in annual plant growth and human diets. Human tissue incorporates the contemporary ^14^C signature of individuals’ food at the time of synthesis. Years later, specific biomolecules can be isolated and measured for ^14^C content to establish carbon turnover or lack thereof, as in the cellulose of tree rings.

Extensive aboveground testing of nuclear weapons doubled the atmospheric level of ^14^C between the mid-1950s and the implementation of the Limited Test Ban Treaty in 1963 (see Figure 1). This pulse rapidly and evenly distributed throughout the Earth’s atmosphere as ^14^CO_2_ [31,32]. Since the test ban, atmospheric radiocarbon concentrations have been decreasing exponentially as atmospheric CO_2_ mixes into large terrestrial and marine carbon reservoirs. Known as the “bomb pulse,” this phenomenon is well characterized with an extensive high-resolution record [33-37]. Atmospheric ^14^C levels from the bomb pulse closely correlate with the ^14^C fingerprint found within dietary components from that given year, and moreover, it has been confirmed that the annual ^14^C averages in food matched those found in human tissue [38]. In a literal sense, anyone alive during this era unwittingly took part in a long-term radiocarbon tracer study where clinically safe levels of ^14^C were incorporated into all proteins, DNA, and cellular structures, thus enabling researchers to date the synthesis of a variety of biomolecules [39-56]. In this study, we determine the date of synthesis of water-soluble and water-insoluble proteins from the human eye lens to determine the rate of carbon turnover.

## Methods

### Human lens dissection

The study was reviewed by the Lawrence Livermore National Laboratory Institutional Review Board and determined to be exempt. Human eyes were obtained through the University of California Davis Donated Body Program and Sierra Eye and Tissue Donor Services of Sacramento, CA. A total of 16 lenses ranging in age from 42 to 83 years were analyzed in this study. Eye globes were frozen immediately postmortem and remained frozen until the time of dissection. Lenses were carefully extracted from the eye globes and verified to be structurally intact. In lenses obtained before 2009, layers of fiber cells were removed incrementally from their chronologically stratified topology by employing a stirring technique [57-61]. Lenses were allowed to reach room temperature and then placed in a 250 ml glass beaker with 100 ml deionized water (DDH_2_O) and a small magnetic stir bar atop a magnetic stirring plate. The layers of cells were slowly peeled away concentrically in the stirred water. Visual inspection determined when enough cells had been removed to yield sufficient carbon for isotope analysis. All of the water containing the peeled cells was removed by pipette, along with two small rinses of DDH_2_O. The remaining portion of lens was carefully transferred to a second 250 ml beaker containing a clean stir bar and 100 ml of fresh DDH_2_O. The process was repeated until the entire lens had been peeled into the water. Lenses obtained after 2009 were frozen in liquid nitrogen and shaved from the outside toward the center until the nucleus was reached. The innermost slices (~500 μm) were collected and analyzed. The center slices primarily consisted of the lens nucleus although the edge of the slices contained some younger cells. Isolated fiber cells were lysed by freeze/thaw cycles and centrifuged at 20,000 × *g* for 30 min at 4 °C to separate the water-soluble and water-insoluble components of the cells. The samples processed after 2009 were further purified using molecular weight spin filters (Sartorius Vivaspin 3000 MWCO, Goettingen, Germany) to remove small molecules. The filters were initially rinsed with 5 ml of 18.2 MΩ water to remove preservatives from the filters, and then the samples were loaded onto the filters following the manufacturer’s directions. The samples were rinsed an additional 3–5 times with 18.2 MΩ water to remove residual small molecules and then concentrated to less than 500 μl for ^14^C analysis.

### ^14^C analysis

Isolated fractions of water-soluble and water-insoluble proteins were analyzed for ^14^C/C concentration by accelerator mass spectrometry (AMS). All AMS analyses were performed blind to the age and origin of the sample. Water-soluble and water-insoluble protein fractions were lyophilized to dryness and transferred to quartz combustion tubes. NIST SRM 4990C (Oxalic Acid II) and IAEA C-6 (sucrose) were dissolved in water and co-lyophilized with the samples as controls to monitor carbon contamination during drying. Excess copper oxide was added to each dry sample, and the tubes were brought to vacuum with a turbo pump station and sealed with an H_2_/O_2_ torch. The tubes were placed in a furnace set at 900 °C for 3.5 h to combust all carbon to CO_2_). The evolved CO_2_ was purified, trapped and reduced to graphite in the presence of iron catalysts in individual reactors [62,63]. All ^14^C/C measurements were completed with graphite targets, which were analyzed at the Center for Accelerator Mass Spectrometry at Lawrence Livermore National Laboratory on the HVEE FN-class AMS system.

A δ^13^C fractionation correction of –20±2 was used for all samples based on the measurements of selected sample splits of large CO_2_ samples. Corrections for background contamination introduced during sample preparation were made following standard procedures [64]. All data were normalized with six identically prepared NIST SRM 4990B (Oxalic Acid I) standards. We used NIST SRM 4990C, IAEA C-6, and TIRI wood [65] as quality control secondary standards to monitor spectrometer performance. The measurement error was determined for each sample and ranged between ±2–10‰ (1 standard deviation).

^14^C/C concentrations were reported using the F^14^C nomenclature for reporting post-bomb data defined in Equation 2 of Reimer et al. [66], which is the enrichment or depletion of ^14^C relative to the oxalic acid standard normalized for isotope fractionation. All ^14^C data in Table 1 and Table 2 were reported as F^14^C and decay corrected ∆^14^C following the convention of Stuiver and Polach [67]. This convention, established for reporting radiocarbon data in chronological and geophysical studies, was not developed to deal with post-bomb data. ∆^14^C was calculated using the following formula:

**Table 1:** Lens nuclei ^14^C Data

Year of Death	Age (y)	Water-soluble fraction				Water-insoluble fraction			
		F^14^C	±*^a^*	∆^14^C	±*^a^*	F^14^C	±*^a^*	∆^14^C	±*^a^*
2005	42	1.2942	0.0051	285.5	5.1	1.2217	0.0091	213.4	9.1
2005	65	1.1018	0.0042	94.5	4.2	1.0319	0.0039	25.1	3.9
2006	73	1.1027	0.0040	95.2	4.0	1.0158	0.0038	8.9	3.8
2006	53	1.0780	0.0039	70.7	3.9	1.0333	0.0046	26.3	4.6
2007	62	1.0298	0.0038	22.8	3.8	1.0002	0.0043	-6.6	4.3
2007	62	1.0623	0.0040	55.0	4.0	0.9946	0.0038	-12.3	3.8
2007	61	1.0581	0.0039	50.9	3.9	0.9557	0.0035	-50.9	3.5
2007	61	1.1038	0.0038	96.2	3.8	0.9980	0.0038	-8.9	3.8
2007	80	1.0325	0.0042	25.4	4.2	0.9934	0.0039	-13.4	3.9
2007	80	1.0675	0.0041	60.1	4.1	1.0017	0.0040	-5.1	4.0
2007	74	1.0385	0.0040	31.4	4.0	0.9890	0.0043	-17.7	4.3
2010	83	1.0229	0.0071	15.4	7.1	0.9850	0.0044	-22.3	4.4
2010	70	1.1045	0.0039	96.5	3.9	1.0521	0.0035	44.5	3.5
2010	82	1.0335	0.0036	25.9	3.6	1.0252	0.0031	17.7	3.1
2010	83	1.0759	0.0039	68.1	3.9	1.0100	0.0033	2.7	3.3
2010	83	1.1166	0.0039	116.6	3.9	1.0297	0.0037	22.3	3.9

*^a^*Uncertainties are ± 1 s.d.

**Table 2:** ^14^C Data from Layers Peeled from Human Lenses

53 year old										
1953–2006		Water-soluble					Water-insoluble			
Layer		F^14^C	±	∆^14^C	±		F^14^C	±	∆^14^C	±
1		1.0830	0.0038	75.7	3.8		1.0982	0.0036	90.8	3.6
2		1.1363	0.0038	128.6	3.8		1.1825	0.0044	174.5	4.4
3		1.2791	0.0051	270.4	5.1		1.3658	0.0052	356.6	5.2
4		1.2585	0.0053	250.0	5.3		1.3063	0.0059	297.5	5.9
5		1.1463	0.0041	138.6	4.1		1.0815	0.0045	74.2	4.5
6		1.1689	0.0048	161.0	4.8		na.	na.	na.	na
7		1.0780	0.0039	70.7	3.9		1.0333	0.0046	26.3	4.6

80 year old										
1926–2007		Water-soluble					Water-insoluble			
Layer		F^14^C	±	∆^14^C	±		F^14^C	±	∆^14^C	±
1		1.1245	0.0039	116.8	3.9		1.1836	0.0042	175.4	4.2
2		1.1918	0.0040	183.6	4.0		1.2165	0.0041	208.1	4.1
3		1.0686	0.0036	61.2	3.6		1.0439	0.0033	36.7	3.3
4		1.0328	0.0037	25.7	3.7		0.9949	0.0041	−11.9	4.1
5		1.0251	0.0040	18.1	4.0		0.9999	0.0033	−7.0	3.3
6		1.0328	0.0034	25.7	3.4		0.9988	0.0033	−8.0	3.3

65 year old										
1939–2005		Water-soluble					Water-insoluble			
Layer		F^14^C	±	∆^14^C	±		F^14^C	±	∆^14^C	±
1		1.1541	0.0044	146.4	4.4		1.2040	0.0065	196.0	6.5
2		1.1542	0.0051	146.5	5.1		1.2019	0.0049	193.9	4.9
3		1.2738	0.0049	265.4	4.9		1.2914	0.0051	282.8	5.1
4		1.2578	0.0047	249.4	4.7		1.2001	0.0058	192.1	5.8
5		1.1018	0.0042	94.5	4.2		1.0319	0.0039	25.1	3.9

Layer numbers are incremental, starting from the outer cortex/capsule (layer 1) and progressing inward to the nucleus. Uncertainties are ± 1 s.d. Sample marked na was not available due to loss during processing.

∆^14^C=1000 * {F^14^C * exp[λ*(1950 - y)] –1} (1)

where λ=1/8267 year^−1^ and y=year of measurement after 1950.

### Protein identification of water-insoluble fractions

Water-insoluble protein samples were suspended in Rapigest (Waters, Bedford, MA), reduced and alkylated, according to the Rapigest standard protocol, and digested with sequencing grade trypsin per the manufacturer’s recommendations (Promega, Madison, WI). Protein identification was performed using a Paradigm LC system (Michrom Bioresources, Inc., Auburn, CA) coupled to a LTQ linear ion trap-quadropole mass spectrometer (Thermo-Fisher, San Jose, CA) through a Michrom nanospray source (Michrom Bioresources, Inc.). Peptides were loaded onto a Michrom nanotrap (Michrom Bioresources, Inc.) and were then eluted and separated by a Michrom 0.2 × 150 mm column packed with Michrom Magic C18 reverse phase material (Michrom Bioresources, Inc.). Peptides were eluted using a 70-min gradient of 2–80% solvent B [buffer A=99.9v% DDH_2_O and 0.1v% formic acid; solvent B=95v% acetonitrile, 4.9v% DDH_2_O, and 0.1v% formic acid] at a flow rate of 2 μl/min. The top 10 ions in each survey scan were subjected to automatic low energy collision-induced dissociation.

For database searching, product mode mass spectrometry/mass spectrometry (MS/MS) spectra were extracted and charge state deconvoluted by BioWorks version 3.3 (Thermo-Fisher, San Jose, CA). Deconvoluted MS/MS spectra were analyzed using X! Tandem software (version 2006.04.01.2; The Global Proteome Machine Organization, Open Source Initiative, East Palo Alto, CA). For fragment recognition, iodoacetamide, a derivative of cysteine, was specified in Mascot (Matrix Science, Inc., Boston, MA) and X! Tandem as a fixed modification, and the oxidation of methionine was specified as a variable modification.

### Protein identification of water-soluble fractions

Protein samples were dissolved in Milli-Q water (EMD Millipore Corporation, Billerica, MA) and then filtered with Whatman 0.45 μm NYL filter (GE Healthcare Life Sciences, Piscataway, NJ). Protein identification was performed based on the exact molecular mass measurements using high-resolution liquid chromatography mass spectrometry data. Exact mass measurement experiments were performed in positive ion mode on a Micromass/Waters LCT, an orthogonal acceleration time-of-flight mass spectrometer (Micromass, Manchester, UK) configured with a dual sprayer electrospray ion source, a standard Z-spray electrospray ionization source, and a lock-spray source, which samples analyte and reference ions independently. The output analog signal was digitalized using a 4 GHz time-to-digital converter.

A Waters Alliance 2795 (Bedford, MA) high-performance liquid chromatography system equipped with a Magic C18, 5 μm, 2.0 × 150 mm column (901–61221–00; Michrom Bioresources, Inc., Auburn, CA) was used for sampling and solvent delivery. The column was equilibrated for 4 min with 100% solvent A at an ambient temperature using a flow rate of 0.350 ml/min. The mobile phase used the following gradients: 0 min, 100% A; 5 min, 60% B; 25 min, 100% B; 25–30 min, 100% B. Mobile phase solvents expressed in volume percentages were the following: A: DDH_2_O 99.9%, trifluoroacetic acid 0.1%, (Sigma-Aldrich, St. Louis, MO; spectrophotometric grade, 99+); B: methanol 99.9%, trifluoroacetic acid 0.1%. A Waters 996 photodiode array (PDA) detector was used for ultraviolet-visible signal detection over a wavelength range of 210–650 nm with a resolution of 1.2 and sampling rate of 1.0 spectrum/s.

In terms of the mass spectrometry conditions, the ion source parameters were as follows: capillary voltage 3,200 V, sample cone voltage 35 V, extraction cone voltage 3 V, source temperature 110 °C, and desolvation temperature 350 °C. The transfer optics settings were as follows: rf lens 375 V, rf dc offset-1 was set to 4.0 V, rf dc offset was set to 3.0 V, aperture 1.0 V, acceleration 180.0 V, focus 4.0 V, and steering 0.0 V. The analyzer settings were as follows: the resolution was set to 7,500 at m/z 800 Th, multichannel plate detector 2,650 V, ion energy 32.0 V, tube lens 18.0 V, grid-2 42.0 V, time-of-flight tube 4,578.0 V, and reflectron 1,813.0 V. The cone gas and desolvation gas were set to 15 and 660 L/h, respectively. The lock spray parameters were identical to the sample setting parameters. The lock spray sampling frequency mode was set to 5.

The data files were acquired in continuum mode, and the spectra were stored from m/z 100 to 2,300 with a 2.3 s scanning cycle consisting of a 2.2 s data scan and a 0.1 s inter-scan delay. The pusher cycle time was set to 75 μs. The time-of-flight calibration and lock mass setup used a Lt_eff_ (effective length of the flight tube) value of 1,124.8000 using the molecular ion signal of the leucine-enkephalin at m/z=556.2771 Th. System calibration was performed using Poly-D-L-alanine (P9003, Sigma Aldrich), and a 50 ng/ml solution of leucine-enkephalin (L9133, Sigma Aldrich) was infused at 5 μl/min into the lock spray (positive ion lock mass: 556.2771 Th) using an ISCO μLC-500 microflow pump (Teledyne Isco, Inc., Lincoln, NE). Savitsky-Golay smoothing using a ± 4 channel window was repeated twice and centered using the center at the 80% peak height to obtain accurate masses. After data acquisition and signal averaging, the spectra were individually corrected relative to the leucine-enkephalin lock mass calibration mass via standard procedure (MassLynx 4.0 SP2 software, Waters/Micromass, Manchester, U.K.).

To obtain accurate mass measurements, the raw data were corrected with lock mass in all cases. To identify given protein components, a library was created with all the known crystallins and possible modifications as independent variables [68]. MassLynx 4.0 SP2 software was used for instrument control, data acquisition, and data evaluation.

## Results

### ^14^C levels of water-soluble and water-insoluble proteins from lens nuclei

Lens nuclei isolated from 16 lenses from 12 different subjects were separated into water-soluble and water-insoluble components and analyzed for ^14^C levels. Table 1 presents the ^14^C concentration for these 16 nuclei and is reported in the F^14^C and Δ^14^C nomenclature. For every sample examined, the water-soluble fraction possessed a F^14^C value greater than the water-insoluble fraction. Many of these F^14^C values were only present in the atmosphere after the aboveground testing of nuclear weapons occurred, which was well after the birth date of many of these subjects. The water-insoluble proteins maintain the lower levels of ^14^C present around the time of birth of the fetal nuclei. The shaved lenses had some younger cells from the outer edges of the slices, however, and consequently had a slightly higher F^14^C than the peeled lenses. The difference in F^14^C between water-soluble and water-insoluble paired samples was similar for each dissection method.

To estimate the amount of turnover that occurred in the water-soluble protein fraction compared to the water-insoluble fraction, the difference in log isotope ratios for the two fractions was examined. For the data from the lens nuclei of donors born before the start of the bomb pulse (i.e., before 1955) in Table 1, the difference in the log isotope ratios (IR), log(IR_soluble_) - log(IR_insoluble_)=log(IR_soluble_/IR_insoluble_), was always positive, and the one sample Student *t* test had a *t*-statistic of 8.6689 for 14 degrees of freedom for a p value of 0.00000053. The 95% confidence interval for the difference was (0.0453, 0.0717) indicating that the isotope ratio of the water-soluble fraction was at least 4–5% larger than that of the insoluble fraction.

### ^14^C levels of water-soluble and water-insoluble proteins from layers of topologically stratified fiber cells from lenses

**Figure 2:**
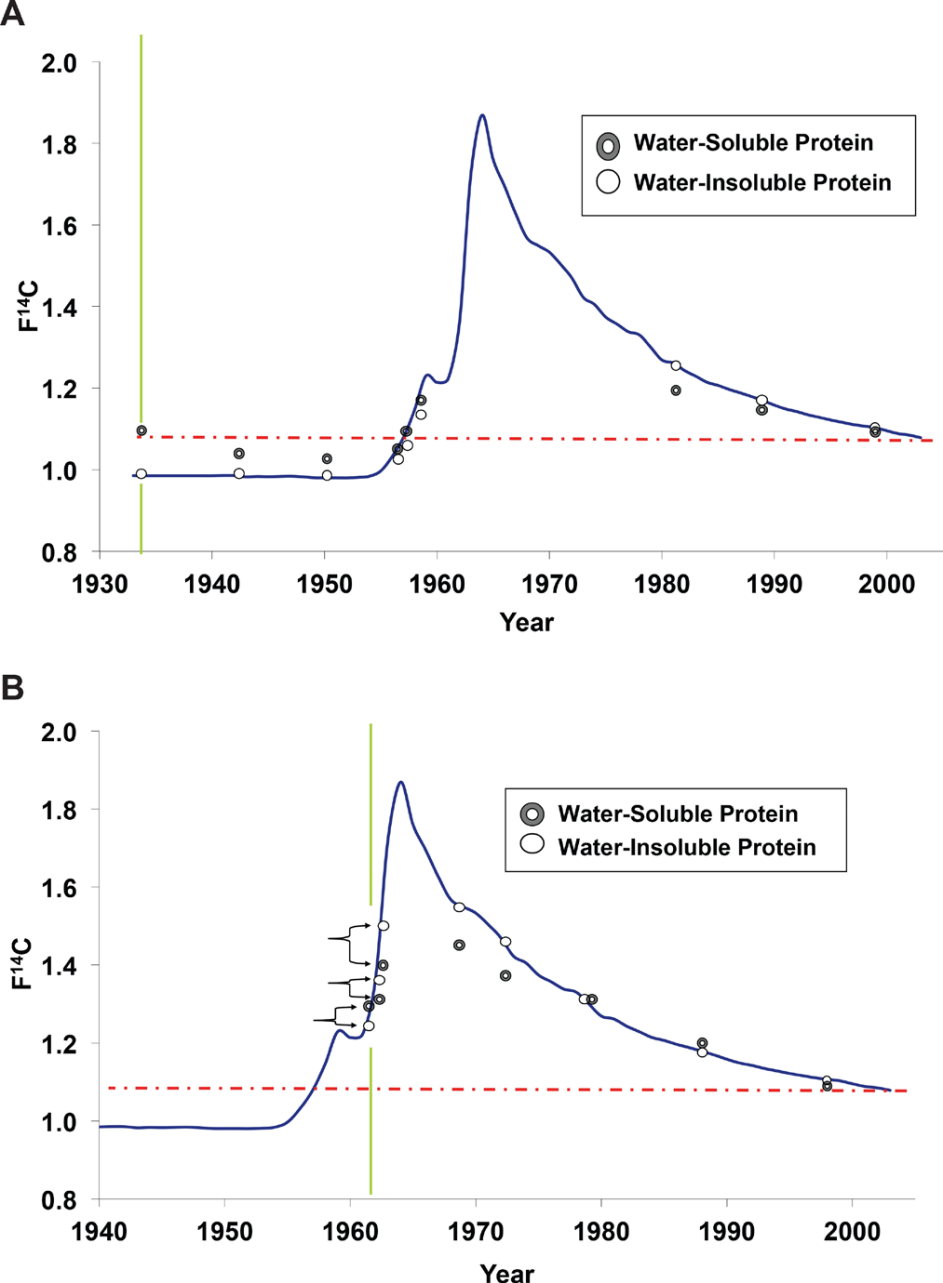
Water-soluble and water-insoluble proteins from the same cells possess different ^14^C signatures. Fiber cells from human cadaver lenses were peeled away in concentric layers to step back in time from the periphery (youngest) to the embryonic nucleus (oldest). Each fraction containing multiple layers of cells was centrifuged to separate the soluble proteins (crystallins exclusively) from the insoluble proteins (membrane, cytoskeleton, precipitated or insoluble crystallins). Data pairs along the atmospheric record (solid black line) represent the water-soluble and water-insoluble fractions from the same cohort of cells. Vertical lines denote the year of birth for each subject born in 1933 (**A**) and 1962 (**B**). The dashed horizontal line denotes the contemporary ^14^C concentration in the atmosphere at the time of death. The insoluble fraction exhibits little or no carbon turnover and is correlated to the average “birth date” of the group of cells. The insoluble fractions of the inner layers of the subject born in 1933 (**A**) do not contain any measurable new carbon while the corresponding soluble fractions are skewed by the addition of more recent carbon in both subjects (**A**, **B**). The younger carbon in the soluble crystallins provides direct evidence of protein turnover. Error bars are smaller than the symbols, averaging ±0.005.

Layers of topologically stratified fiber cells from adult human cadaver lenses were removed incrementally using the water peeling method, enabling the isolation of similarly aged populations of fiber cells. The water-soluble and water-insoluble proteins were isolated from these cells, and AMS was used to determine the ^14^C levels present in the water-soluble and water-insoluble proteins isolated from each of these cell layers. Figure 2 shows the ^14^C/C concentration reported in F^14^C units of the water-soluble and water-insoluble proteins isolated from the subjects born in 1933 (A) and 1962 (B), and where they fall along the atmospheric record for ^14^C. For both donors, the F^14^C for the water-insoluble proteins from the innermost (oldest) layer corresponds to the atmospheric level of ^14^C present in the atmosphere at the time of the subjects’ date of birth. The ^14^C level in the insoluble protein fraction relates to the average birth date of the cells in that sample and was used to map the water-soluble data to the appropriate location on the bomb curve (i.e., the ^14^C level of the water-insoluble fraction anchors the chronological location on the bomb curve). Along the curve, the ^14^C level of the water-soluble fraction from each sample is plotted above or below the established average birth date as determined by the water-insoluble fraction of the whole cells from which it was derived.

For the donor born in 1933 (Figure 2A), the inner six (oldest) layers display water-soluble fractions with higher F^14^C values than their water-insoluble counterparts. The higher F^14^C values correspond to the water-soluble fractions containing younger carbon on average than their water-insoluble counterparts. The three outer (youngest) layers possess F^14^C values for the water-soluble protein lower than for the water-insoluble protein, which again corresponds to the water-soluble fractions containing younger carbon on average compared to their water-insoluble counterparts. The drift of the ^14^C concentration away from the levels established on the cells’ birthday is due to the inclusion or incorporation of newer carbon over the course of the donor’s lifetime. New carbon incorporation in cells formed before the bomb pulse is not perceivable until 1955 and is always observed as an increase in ^14^C/C concentration above the atmospheric record. New carbon incorporation for cells born after the peak of the bomb pulse in 1963 is always observed as a decrease in ^14^C/C concentration. The offset in the radiocarbon signature between the water-soluble and water-insoluble fractions allows for the quantification of the extent to which carbon turnover has occurred. For the donor born in 1962 (Figure 2B) when aboveground testing of nuclear weapons was still occurring, cells from infancy were able to experience years of both higher or lower atmospheric levels of ^14^CO_2_. The addition of new cellular carbon might have initially increased and then later decreased the average ^14^C/C concentration with respect to its concentration at birth.

Table 2 lists the ^14^C/C concentration reported in F^14^C and Δ^14^C units for the water-soluble and water-insoluble proteins from layers of topologically stratified fiber cells from three additional adult human cadaver lenses. The layers are numbered with “1” representing the outermost layer. In the case of the lens of the donor born in 1953, aboveground testing of nuclear weapons had started, but the atmosphere did not reflect the anthropogenic ^14^C until 1955. Similar to the lens depicted in Figure 2B, the cells born when the donor was a child during the period when testing was occurring could have experienced later years of both higher or lower atmospheric levels of ^14^CO_2_. The addition of new cellular carbon might have initially increased and then later decreased the average^14^C/C concentration of proteins.

The other two lenses in Table 2 are from individuals born before the bomb pulse. The inner layers of the lenses contain the oldest synthesized proteins, yet the F^14^C for the water-soluble fraction is greater than that of the water-insoluble proteins, which indicates the incorporation of younger (bomb-elevated) ^14^C in the cells born before the atmospheric testing of nuclear weapons. All carbon incorporated into the existing lens cells after 1955 has a higher F^14^C than that in the water-insoluble fractions of those cells. The outer layers of the lenses contain the younger cells formed after 1963. The carbon added to these cells after their formation will always have a lower F^14^C. In the cells born after the peak of the bomb pulse (i.e., after 1963), the water-soluble fractions possessing a lower F^14^C than the paired water-insoluble fractions indicate new carbon incorporation in those cells.

### 3.3 Proteins identified in the water-insoluble fractions

The proteins present in the water-insoluble fraction of the lens were identified using liquid chromatography (LC)-MS/MS. Table 3 presents the proteins identified in the water-insoluble fractions from the cadaver lenses. Two major categories of proteins were identified in the water-insoluble fractions: (1) crystallins and (2) membrane and cytoskeletal proteins. The method of protein identification included digestion and solubilization for liquid chromatography, which also causes modifications. The water-insoluble fractions had much less mass than the water-soluble fractions. Material was not always available for protein mass spectrometry for small water-insoluble samples because all the carbon was needed for isotope analyses. The list of modifications of the crystallins in Table 3 is not exhaustive but includes the most common, identified modifications.

**Table 3:** Identified proteins of the water-insoluble fractions

Water-insoluble crystallins	Water-insoluble membrane and cytoskeletal proteins
α Crystallin A chain	Filensin
α Crystallin B chain	Phakinin
β Crystallin A2	Spectrin
β Crystallin A3	Actin
β Crystallin A4	Tubulin
β Crystallin B1	Fibrillin
β Crystallin B2	Keratin
β Crystallin B3	Vimentin
γ Crystallin S	Aquaporin-0 (AQP0)
γ Crystallin B	
γ Crystallin C	
γ Crystallin D	

Water-insoluble crystallins with modifications	
α Crystallin A chain	acetylation
α Crystallin A chain	2 x acetylation
α Crystallin A chain	Cysteine crosslink
α Crystallin A chain	Cysteine crosslink + acetylation
α Crystallin B chain	acetylation
α Crystallin B chain	acetylation + (sulphonation or phosphorylation)
α Crystallin B chain	acetylation + 2 x (sulphonation or phosphorylation)
α Crystallin B chain	sodiation

### Proteins identified in the water-soluble fractions

Proteins present in the water-soluble fraction of the lens were identified using high-resolution liquid chromatography mass spectrometry. Table 4 presents the proteins identified in the water-soluble fractions from the cadaver lenses. The proteins identified in the water-soluble fractions were all crystallins and harbored a variety of modifications. The list in Table 4 contains the majority of proteins, accounting for the carbon mass associated with the isotopic measurements, but it is not an exhaustive list of all proteins detected.

**Table 4:** Identified proteins of the water-soluble fractions

Protein	Observed Mass (Da)	Modification
α Crystallin A chain	19932.8	Sodiation
	19950.2	Acetylation
	19906.2	Cysteine crosslink
	19949.8	Cysteine crosslink + Acetylation
α Crystallin B chain	20180.4	Sodiation
	20200.2	Acetylation
	20221.6	Sodiation + Acetylation
	20242.3	2 x Acetylation
	20279.6	Acetylation + Sulphonation or phosphorylation
	20360.8	Acetylation + dual Sulphonation and or phosphorylation
γ Crystallin C chain	20878.8	none
γ Crystallin S chain	21005.0	none

## Discussion

The transport and circulation of water, electrolytes, and small molecules within the lens is well documented and believed necessary for lens health [1,13-15,26]. This circulation is both transverse, spanning layers of cells of different ages, and concentric, among layers of cells of the same age. A large molecule diffusion pathway, distinct from the gap junctions, has also been described in the literature [27-29]. This pathway is found primarily between cells of the same age and has been demonstrated using fluorescent proteins in mice and chickens. The transverse transport of proteins among cells of different ages is especially low over short spans of observation [27-29]. The transport of young carbon in protein synthesized by cortical fiber cells to the deeper nuclear fiber cells via the large molecule diffusion pathway may account for the observed 4–5% difference in ^14^C/C observed between water-soluble and water-insoluble proteins of the lens nuclei.

The first direct evidence of in vivo carbon transport and turnover in adult human nuclear fiber cells was identified using the chronologically variable level of ^14^C introduced to the atmosphere by aboveground nuclear weapons testing from 1955 to 1963 [35-37]. This turnover occurs in the water-soluble proteins of the fiber cells. Our key finding is the discovery of an influx of new carbon within even the oldest fiber cells of the human eye lens. This water-soluble carbon is from a protein fraction identified as containing crystallin protein in the mass range of 19.5–21.5 kDa. This is the first identification of in vivo carbon turnover in protein from human nuclear fiber cells and contradicts the prevailing paradigm that carbon turnover does not occur in this region of the lens. In a study exploring the lens as a tissue for forensic dating, lens nuclei were surgically extracted and dated as a whole tissue [49]. Although the authors stated there was no protein turnover, they did not isolate the proteins and their data also clearly shows new carbon in the lens nuclei [49].

It is reasonable that peptide or amino acid transport mechanisms are bidirectional, so carbon can be transported radially in both directions. New carbon can move from outer young cells to inner old cells, and old peptides can move from inner old cells toward younger cells on the outer layers of the lens. Based on our limited data, there is a hint that peptides (or amino acids) are recycled from older regions of the lens back into the cortex. The 1989 data pair from the healthy 42-year-old lens in Figure 2B exhibits a level of water-soluble ^14^C that is elevated above subsequent atmospheric levels. The likely source of this material would be from deeper within the tissue itself or variability in the cell layers removed in the peeling dissection method. This recycling, not readily observable in the older lenses we examined, may be evidence that transport of materials between the cortex and the nucleus attenuates with age, which agrees with prior findings [4,16].

The younger carbon measured in the lens nuclei might also be a product of protein synthesis or repair from transported smaller molecules. It is difficult, however, to devise a mechanism by which protein synthesis could occur in the nucleus. Yet our finding of younger carbon in water-soluble proteins of the nucleus is consistent with a study where poly(A)^+^mRNA was recovered and purified from bovine nuclear fiber cells and found to synthesize a protein profile distinct from that expressed in the lens cortical cells [69]. The profile was comprised predominantly of crystallin proteins and was distinctly lacking in the expression of the higher molecular mass membrane and cytoskeletal proteins. Our data is consistent with these findings and suggests that there could be a mechanism of small molecule transport combined with onsite synthesis or repair of crystallin proteins in nuclear fiber cells. Our findings additionally show that human lens nuclei do not behave in a manner consistent with a syncytium, as observed in chickens and mice [27,28]. Had the lens nuclei behaved as a true syncytium, the nucleus would have exhibited a significantly more homogenous distribution of ^14^C.

Although recycling of carbon within the lens complicates our ability to accurately quantify the total degree of carbon turnover in these cells, models can be constructed to estimate the degree of carbon turnover by using the atmospheric ^14^C record and the paired data. Carbon turnover implies the replacement of old carbon with new while keeping the total carbon inventory constant. The cycling carbon could be contained in whole proteins, peptides, amino acids, or other carbonaceous compounds. Models were constructed to account for the turnover of carbon.

**Figure 3:**
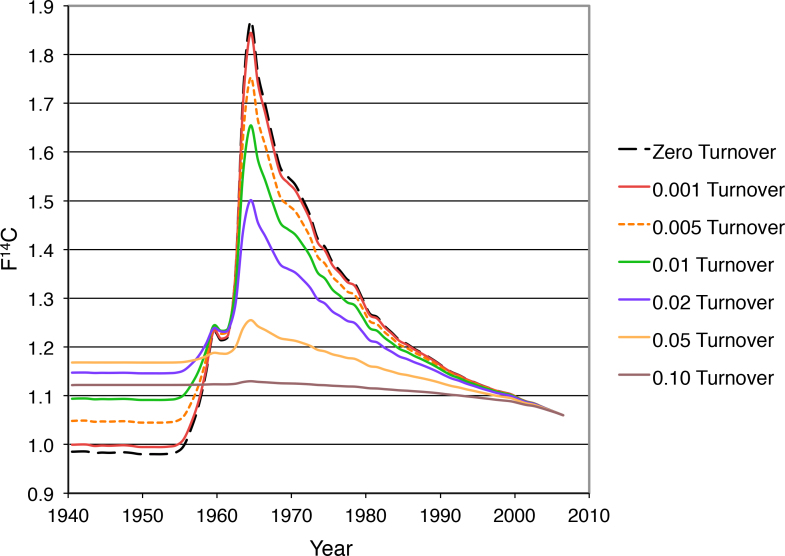
Annual carbon turnover changes the shape of the ^14^C bomb curve. Carbon turnover in a macromolecular sample flattens the pulse of ^14^C as the rate of turnover increases. When turnover reaches 0.10 (10% annually) the pulse is almost completely flattened. If turnover is less than 0.001 (0.1% annually), molecules formed before 1955 are elevated in ^14^C by about 2%, but turnover is difficult to detect in molecules formed after the onset of the pulse. The differences in ^14^C between water-soluble and water–insoluble proteins fit a model suggesting ~0.005-0.01 turnover (0.5-1% annually) of carbon in water-soluble protein.

One model assumes the annual replacement of carbon with no physiological preference in which carbon atoms are replaced (i.e., any carbon atom in a population was equally likely to turnover each year). For example, if a tissue has an annual carbon turnover of 1%, 99% of the carbon is unchanged and 1% of the carbon gets the next year’s F^14^C ratio from the published annual growing season average (zero turnover). The new total F^14^C is calculated, and the process is repeated annually to produce the curves with different turnover rates (Figure 3). Carbon from subjects born before 1955 always possesses an increased F^14^C compared to their birth level if molecular turnover occurred during the time of the bomb pulse. Carbon from subjects born on the rise of the pulse (i.e., 1955–1963) can exhibit an increased or decreased F^14^C depending on the rate of turnover and where they fall on the curve. Carbon from subjects born after the pulse always exhibits a decreased F^14^C if molecular turnover occurred because the newer carbon always has a lower F^14^C after the peak in 1963. If a small population of carbon molecules undergoes turnover in a primarily static pool, the deviation from zero turnover can be small [70]. The offset between paired protein samples in Figure 2A is consistent with a 1–2% annual turnover of carbon between synthesis and the date of death. Using this constant annual turnover model, we estimate that the water-soluble carbon turns over at a rate of ~1% per year while the annual turnover of insoluble protein carbon is 0.0–0.1% (Figure 3). This nominal shift of water-insoluble ^14^C may be due to formerly soluble crystallins from a more modern time becoming insoluble after modification or oxidative damage, or perhaps precipitation (rather than turnover) since the water-insoluble carbon inventory increases.

The development of the cortical barrier makes the lifetime turnover scenario unlikely, however, so another model, which assumed constant annual turnover during the first 40 years of life followed by complete cessation, was examined. The 4–5% elevation of water-soluble F^14^C compared to its paired water-insoluble fraction is consistent with a 0.5–1.0% annual carbon turnover exclusively during the first 40 years of life of the pre-1955 lens nuclei listed in Table 1. The 42-year-old lens from Table 1 does not fit this turnover rate as it needs ~2–3% annual carbon turnover for 40 years to fit the data to the ^14^C record. This higher rate does not fit any of the other lenses, however, and suggests that the rate of new carbon incorporation likely varies with age.

The observed level of ^14^C in the water-insoluble proteins for each layer isolated is consistent with the lack of new membrane/cytoskeletal protein incorporation and suggests that the insolubilized (formerly soluble) crystallin protein observed in the insoluble fractions is a relic from the original cellular material. These proteins preserve their original levels of ^14^C for over 50 years and serve as internal controls for determining the birth date of the population of cells from which they originate. The absence of bomb carbon in the nuclei of peeled lenses demonstrates that our dissection protocols yielded cellular material in a uniform fashion without significant mixing of the older and younger regions of the lens. Exhibiting minimal (if any) turnover, the ^14^C level of water-insoluble protein can provide an approximate “birthday” of the parent cells through correlation with the atmospheric ^14^CO_2_ record as depicted in the bomb pulse (Figure 2A). This ascribed date represents an average birthday that spans multiple layers of fibers that were removed concomitantly. With the exception of the nucleus that develops during gestation, we do not have a means to determine the exact time of synthesis of these cells. However, as can be seen in Figure 2B (a lens from a donor born in 1962), we are indeed capable of dating the nucleus to precisely the calendar year in which these cells were formed.

Cataracts are the leading cause of blindness worldwide and are associated with extensive protein oxidation and insolubility. There has yet to be an answer as to why half of the U.S. population over the age of 65 years experiences cataracts [71], but we may be one step closer in our understanding as to why the other half do not develop cataracts. We now know that the incorporation of new carbon into crystallin protein is a regular physiological process of our oldest lens cells. It may well be true that ARN cataracts arise (at least in part) by attenuation or the loss of protein repair or transport mechanisms. If this is indeed the case, therapy for the resuscitation of these mechanisms could alleviate or postpone the need for cataract surgery and save billions of dollars annually for the U.S. Medicare system as well as the healthcare industries of Western Europe and Australia [71].
